# Biologically synthesized black ginger-selenium nanoparticle induces apoptosis and autophagy of AGS gastric cancer cells by suppressing the PI3K/Akt/mTOR signaling pathway

**DOI:** 10.1186/s12951-022-01576-6

**Published:** 2022-10-08

**Authors:** Rongbo Wang, Keum-yun Ha, Sanjeevram Dhandapani, Yeon-Ju Kim

**Affiliations:** Graduate School of Biotechnology, and College of Life Science, Kyung Hee University, Yongin-si, 17104 Gyeonggi-do Republic of Korea

**Keywords:** Selenium nanoparticles, *Kaempferia parviflora*, Gastric cancer, Autophagy, Apoptosis, PI3K/Akt/mTOR

## Abstract

**Background:**

Despite being a promising strategy, current chemotherapy for gastric cancer (GC) is limited due to adverse side effects and poor survival rates. Therefore, new drug-delivery platforms with good biocompatibility are needed. Recent studies have shown that nanoparticle-based drug delivery can be safe, eco-friendly, and nontoxic making them attractive candidates. Here, we develop a novel selenium-nanoparticle based drug-delivery agent for cancer treatment from plant extracts and selenium salts.

**Results:**

Selenium cations were reduced to selenium nanoparticles using *Kaempferia parviflora* (black ginger) root extract and named KP-SeNP. Transmission electron microscopy, selected area electron diffraction, X-ray diffraction, energy dispersive X-ray, dynamic light scattering, and Fourier-transform infrared spectrum were utilized to confirm the physicochemical features of the nanoparticles. The KP-SeNPs showed significant cytotoxicity in human gastric adenocarcinoma cell (AGS cells) but not in normal cells. We determined that the intracellular signaling pathway mechanisms associated with the anticancer effects of KP-SeNPs involve the upregulation of intrinsic apoptotic signaling markers, such as B-cell lymphoma 2, Bcl-associated X protein, and caspase 3 in AGS cells. KP-SeNPs also caused autophagy of AGS by increasing the autophagic flux-marker protein, LC3B-II, whilst inhibiting autophagic cargo protein, p62. Additionally, phosphorylation of PI3K/Akt/mTOR pathway markers and downstream targets was decreased in KP-SeNP-treated AGS cells. AGS-cell xenograft model results further validated our in vitro findings, showing that KP-SeNPs are biologically safe and exert anticancer effects via autophagy and apoptosis.

**Conclusions:**

These results show that KP-SeNPs treatment of AGS cells induces apoptosis and autophagic cell death through the PI3K/Akt/mTOR pathway, suppressing GC progression. Thus, our research strongly suggests that KP-SeNPs could act as a novel potential therapeutic agent for GC.

**Graphical Abstract:**

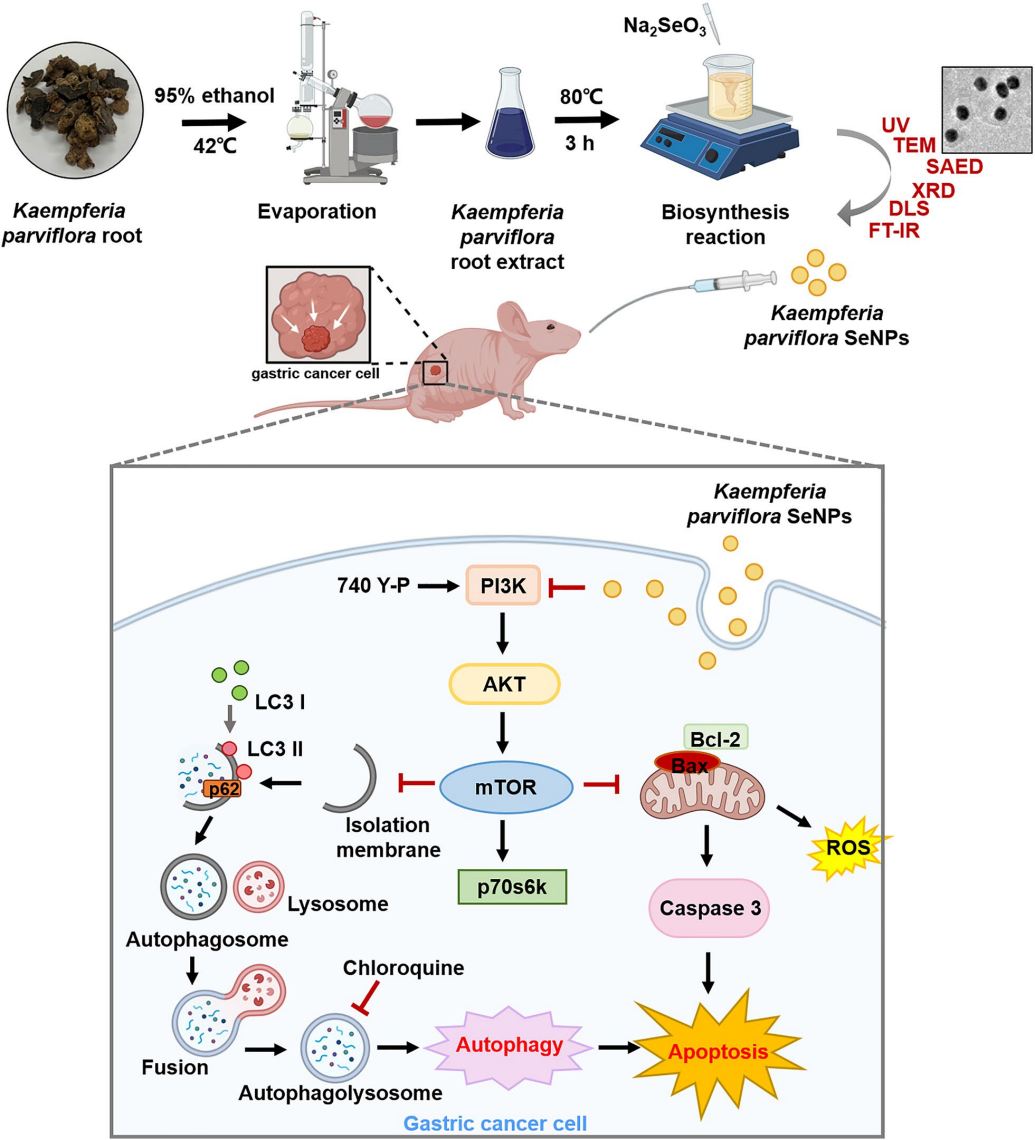

**Supplementary Information:**

The online version contains supplementary material available at 10.1186/s12951-022-01576-6.

## Introduction

Gastric cancer (GC) is a massive global health burden and the second leading cause of cancer mortality [1]. Although novel therapies for GC have been introduced, there have been no significant improvements in the outcomes of new cases. Therefore, it is vital to develop an innovative and effective approach to combat GC. Recently, novel therapeutic applications, such as biosynthetic nanoparticles, have sparked interest in developing a nanoparticle-based drug delivery system that offers benefits over conventional chemical processes in terms of green credentials, energy-efficiency, and cost-effectiveness. Nanoparticles made from gold, silver, copper, zinc, and selenium have proven beneficial in cancer therapy because of excellent pharmacokinetics, specific tumor cell targeting, reduced side effects, and reduced risk of drug resistance [2–5]. In particular, selenium, a physiologically active element, contributes significantly to health maintenance and illness prevention [6]. Recently, numerous studies have focused on the capacity of selenium nanoparticles to demonstrate a variety of activities in vivo, including anticancer [7], antioxidant [8], antibacterial [9], and anti-biofilm effects [10]. Compared to inorganic and organic selenium, selenium nanoparticles (SeNPs) exhibit enhanced bioavailability and lower toxicity [11].

Currently, chemical reduction and radiolysis reduction are the most commonly used nano-selenium production mechanisms [12, 13]. However, the use of chemical reagents or radiation often creates dangerous by-products, leading to significant challenges and health risks. Wadhwani et al. has reported that chemically synthesized SeNPs have shown higher cytotoxicity to normal (non-cancerous) cells than SeNPs synthesized by a greener approach [14]. In addition, bare chemically synthesized SeNPs are highly unstable, often aggregating, and precipitating in aqueous solutions, thereby lowering bioactivity [15]. In contrast, the synthesis of nanoparticles by reduction with plant extracts represents an advance over other methods as it is straightforward, environmentally friendly, easy to scale and screen in a high-throughput manner, and low-cost. Proteins, amino acids, organic acids, vitamins, and secondary metabolites from plant extracts, have been used as reductants and stabilizers in the fabrication of nanoparticles [16]. Research has shown the therapeutic potential of plant based synthesized SeNPs as anti-cancer [17], anti-microbial [18], and antioxidant [19] therapies. In addition, certain selenium-based drug-delivery systems have been created by designing SeNPs with functional ligands in order to deliver pharmaceuticals to specific places. Hence, the environmentally friendly manufacture of SeNPs using plant extracts as the reductant is a burgeoning area of research.

*Kaempferia parviflora* (black ginger), a member of the Zingiberaceae family, is native to Thailand and Laos [20]. *K. parviflora* is a unique medicinal herb that supports a broad range of pharmacological activities, including anti-inflammation [21], antioxidant [22], anti-cancer [23], anti-obesity [24], and anti-cholinesterase effects [25]. The pharmacological agents of *K. parviflora* rhizomes comprise volatile oil, phenolic glycosides, and numerous flavonoids. These functional groups also have the potential to stabilize nanoparticles [26].

To develop new nanomaterials with anticancer effects, we synthesized KP-SeNPs using *K. parviflora* root extract (KP-RE) and selenium salt and characterized its physiochemical properties. Moreover, we identified that KP-SeNPs induce apoptosis and autophagy of AGS gastric cancer cells by suppressing the PI3K/Akt/mTOR pathway. Finally, we found that KP-SeNP-induced apoptosis and autophagy significantly improved GC pathologies seen in the thymus-deficient mice bearing AGS xenografts mouse model.

## Materials and methods

### Materials

Complete cell culture medium, fetal bovine serum (FBS) and penicillin/streptomycin (PS), cDNA synthesis mix kit, qGreen Q-PCR mix kit, protease inhibitor cocktail, phosphatase inhibitor, polyvinylidene fluoride (PVDF) membranes, and enhanced chemiluminescence (ECL) solution were all purchased from Gibco (Grand Island, NY, USA). Sodium selenite (Na_2_SeO_3_), 3-(4,5-Dimethylthiazol-2-yl)-2,5-diphenyltetrazolium bromide (MTT), Hoechst 33258, propidium iodide (PI) solution, Mito-Tracker® Green, TRIsure lysis solution, and RIPA Lysis extraction buffer were obtained from Sigma-Aldrich (St. Louis, MO, USA). The ROS/Superoxide detection assay kit and the HRP/DAB detection IHC kit were purchased from Proteintech Group, Inc (Rosemont, IL, USA). The primers were provided by Macrogen (Seoul, Republic of Korea). The antibodies used in western blot analysis were provided by Santa Cruz Biotechnology (Dallas, TX, USA). Ultrapure water was prepared using water purification system from Human Science (Hanam, Republic of Korea).

### Preparation of plant extract

*K. parviflora* roots were provided by Korea Agricultural Association with authorization number 2019–3. Dried *K. parviflora* roots were crushed into powder. The powder was proceeded to macerated twice in 95% ethanol for 24 h at room temperature (RT). Next, the solution was filtered through filter paper (Whatman International, Ltd., Maidstone, UK) and the filtrate was collected. Evaporation of the ethanoic extract was carried out at 42 °C using the digital water bath (EYELA, Keyland Ct Bohemia, NY, USA) for 48 h until fully evaporated. The extract was then, stored at 4 °C for further experiment.

### Synthesis of KP-SeNPs

The synthesis of KP-SeNPs was optimized by screening different concentrations of Na_2_SeO_3_ and KP-RE, and the reaction circumstances such as pH, temperature, and duration. Sodium selenite and KP-RE were compared in different concentrations of 1–4 mM and 0.5–4 mg/mL, respectively. The pH was controlled from 5 to 11. The temperature was compared from 60–100 °C and the duration was compared from 1–5 h. From the above several reaction conditions, the final optimized conditions included 2 mg/mL of KP-RE in 2 mM Na_2_SeO_3_ solution at pH 9, incubation at 80 °C for 3 h. The synthesized KP-SeNPs were obtained and centrifuged (13,000 rpm, 20 min) to obtain the pure KP-SeNPs. The collected KP-SeNPs were stored at 4 °C until use. Finally, the stabilization of the nanoparticles afforded by the KP-RE was analyzed using a UV–Vis spectrometer (Optizen POP from Daejeon, Korea) and carried out with a double-beam and scanning in the region of 350–750 nm.

### Characterization of KP-SeNPs

To characterize the synthesized KP-SeNPs, transmission electron microscopy (TEM, Tecnai G2 Spirit, FEI Company, USA) at a voltage of 200 kV was used to check morphology, including selected area electron diffraction (SAED), and Energy Dispersive X-ray (EDX) patterns of the nanoparticles. The X-ray diffraction (XRD) of the synthesized KP-SeNPs was performed using a multifunctional X-ray diffractometer (Bruker, Billerica, MA, USA). The nanoparticles were sized by dynamic light scattering (DLS) using a DLS particle analyzer (Otuska Electronics, Shiga, Japan). Fourier-transform infrared (FTIR) spectra of KP-SeNPs and KP-RE was detected by an FTIR spectroscope (PerkinElmer Inc., Waltham, MA, USA) in the range of 4,000–500 cm^−1^ with a resolution of 4 cm^−1^ to analyze the functional groups capped on the surface of the KP-SeNPs.

### Cytotoxicity assay for cancer cells and normal cells

The AGS cells and human keratinocyte cells (HaCaT) were bought from The Korean Cell Line Bank (KCLB, Seoul, Republic of Korea). All cells were seeded in a complete cell culture medium with 10% heat-inactivated FBS and 1% PS. The cells were incubated in 5% CO_2_ atmosphere at 37 °C. An MTT test was performed to check the cytotoxicity of the KP-SeNPs on the cells according to a previously reported method [27].

### Mito-tracker, ROS and Mito-SOX staining

AGS cells were seeded in 6-well plates at the density of 1 × 10^5^ cells/well for 24 h and treated with 200 µg/mL of KP-SeNPs or KP-RE. After 24 h, cells were stained by MitoTracker® Green for 1 h and observed under a fluorescence microscope.

Next, to observe the effect of KP-SeNPs and KP-RE on the production of ROS/Superoxide, AGS cells (1 × 10^5^ cells/well) were seeded in a 6-well plate and treated with 200 µg/mL of KP-SeNPs or KP-RE for 24 h. The intracellular ROS release was detected after incubation with detection reagent for 30 min at 37 °C, following the protocol of ROS/Superoxide detection assay kit. The image was obtained using fluorescence microscope.

### Hoechst and propidium iodide staining

AGS cells were seeded in 6-well plate at the density of 1 × 10^5^ cells/well and maintained for 24 h to investigate the number of apoptotic cells and morphology of the cells. Different doses of KP-SeNPs and KP-RE were added to the AGS cells. 50 µM of cisplatin (Cis) was used as a positive control. After 24 h, the cells were treated with Hoechst 33,258 and PI solution for 30 min at 37 °C. A Leica DM IRB fluorescence microscope (Leica Microsystems, Wetzlar, Germany) was used to take images of the cells.

### Quantitative real-time PCR

The cells were grown until 80% confluence in 100 mm × 20 mm cell culture plates, and then incubated for 24 h after application of the treatment. After washing twice with PBS, TRIsure lysis solution was added to the cells. The obtained RNA was reverse transcribed into cDNA using cDNA synthesis kit, following the manufacturer’s instructions. A q-PCR mix kit was used to perform the qRT-PCR. The list of the primer sequences used are in Additional file 1: Table S1.

### Western blotting

AGS cells were grown to 80% confluence in 100 mm × 20 mm cell culture plates and treated with appropriate amounts of samples. Then, RIPA lysis buffer containing protease and phosphatase was used to lyse the cells. The resultant protein was separated on a sodium dodecyl sulfate–polyacrylamide gel and transferred to PVDF membranes using a gel electrophoresis chamber system (Thermo Fisher Scientific, IL, USA). The blots were blocked with 5% skimmed milk at RT for 50 min. The membranes were incubated with primary antibodies for 3 h and secondary antibodies for 1 h (at RT). To observe and analyze the image of the result, the ECL reagent and Image J software (https://imagej.nih.gov/ij/; NIH, Bethesda, MD, USA) were employed.

### Microtubule-associated protein 1A/1B-light chain 3 (LC3) immunofluorescence staining

AGS cells were cultured to a density of 1 × 10^5^ cells in a 6-well plate and treated accordingly, with 200 µg/mL KP-SeNPs and 5 µM Rapamycin (Rapa) for 24 h. Cells were fixed by 4% para-formaldehyde and permeabilization using 0.1% Triton X-100 for 20 min and blocked with 2% BSA in PBS for 1 h. Next, the cells were incubated with LC3 antibody at RT for 3 h followed by incubation with fluorescein isothiocyanate (FITC)-conjugated secondary antibody for 40 min. Finally, the fluorescence images were captured by a Leica fluorescence microscope (Leica Microsystems, Wetzlar, Germany) and analyzed through using Image J software.

### In vivo xenograft model and experimental design

Animals were approved to proceed with care and used according to the Animal Care and Use Guidelines of Kyung Hee University (KHGASP-21–441). Male nude mice (CAnN.Cg-Foxn1-nu, 4 weeks, 20–22 g) were purchased from Orient Bio (Seongnam, Republic of Korea). One week of adaptation was conducted in a chamber under controlled conditions (23 °C and 50% humidity). After adaptation, AGS cells (1 × 10^7^) were suspended in 100 µL PBS with matrigel (1:1, *v*/*v*) and injected into the right-back of mice. When the average volume of the tumor reached to around 100 mm.^3^, the mice were divided into five groups (n = 5). KP-SeNPs were orally administered at two different doses (5 mg/kg and 10 mg/kg), 5-Fluorouracil (5-FU, 5 mg/kg) was used as a positive control, while mice in the control and control tumor groups were administered with the same amount of 0.9% saline. During treatment, the tumor volume was determined by measuring the length (l) and width (w) and calculating the volume using the formula:

$$Volume = l\times \frac{{w}^{2}}{2}$$

The data was shown as relative tumor volume which was calculated as *V/V*_*0*_, where the tumor volume before the treatment is termed *V*_*0*_. The treatment period was sixteen days. The mice were euthanized after 16-day of administration, and the tumors were dissected and weighed. Tumors and organs, such as the liver and kidney of the mice, were isolated and fixed in 10% neutral buffered formalin for immunohistochemical analysis or hematoxylin and eosin (H&E) staining. The blood of the mice was collected for further experiment.

### Immunohistochemical (IHC) and histology staining

Tumor tissues were promptly fixed in 10% formalin and embedded in paraffin. After that, the slices were dewaxed and stained with hematoxylin and eosin Y (H&E) for histological examination under a light microscope. The IHC labeling was followed according to the instruction of the mouse and rabbit-specific HRP/DAB (ABC) Detection IHC Kit. The stained tissues were observed under fluorescence microscope.

### In vivo toxicity assay

The serum for biochemical analysis was conducted for determining the in vivo toxicity of the samples. The mouse blood was centrifuged (3000 rpm, 10 min) to obtain the serum. The level of alanine aminotransferase (ALT), aspartate aminotransferase (AST), total cholesterol (T-Chol), albumin/globulin (A/G) ratio, triglyceride (TG), glucose (GLU), blood urea nitrogen (BUN), and creatinine (Crea) were checked by a Fuji Dri-Chem analyzer (Fuji Photo Film Co., Osaka, Japan).

### Liquid chromatography–mass spectrometry (LC–MS) analysis of KP-SeNPs

KP-SeNPs (1.0 g) and KP-RE (1.0 g) was mixed with 5% DMSO/MeOH, respectively, and filtered using 0.22 μm syringe filters (ADVANTEC, Tokyo, Japan). The LC analysis was performed on an Ultimate 3000 system (Thermo Scientific, USA) according to previously reported methods [27].

### Statistical analysis

All experiments were performed in triplicate, and the data are expressed as the mean ± standard deviation. Student's t-test was used for statistical comparison between two groups, and the results were considered as significant at *p < 0.05, ** *p* < 0.01, ***p < 0.001.

## Results and discussion

### Physicochemical characteristics of KP-SeNPs

**Fig. 1:**
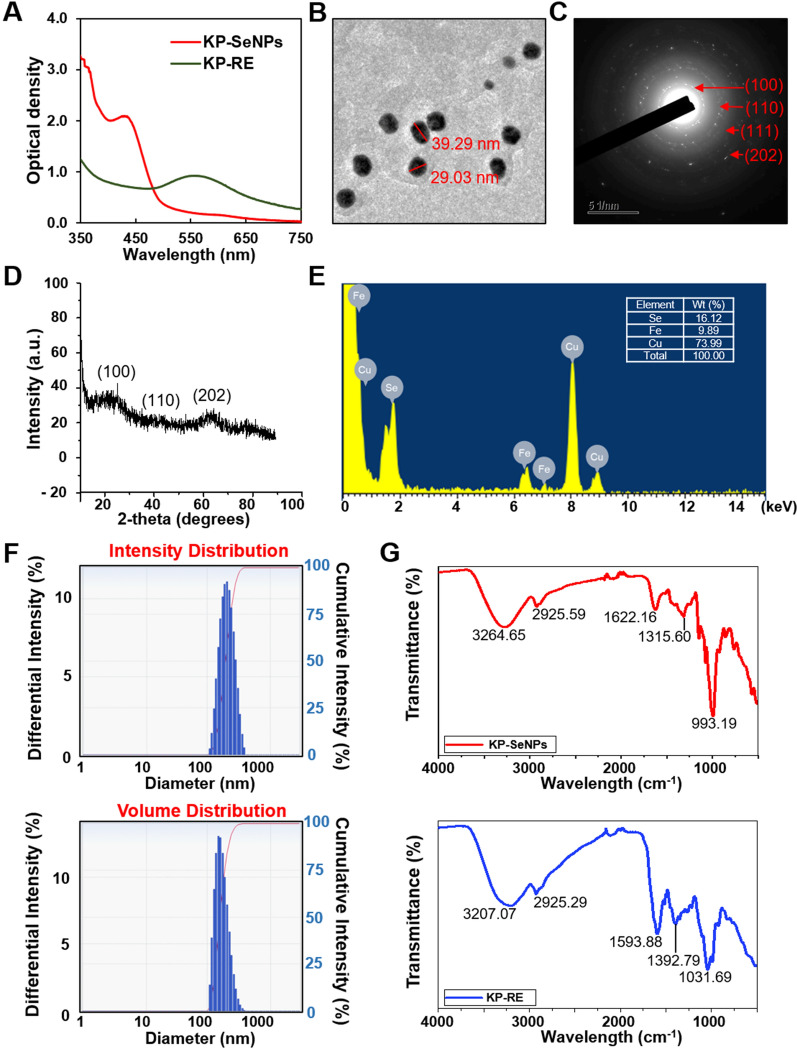
Characterization of KP-SeNPs. **A** UV–Vis spectrum of KP-SeNPs and KP-RE. **B** Transmission electron microscope (TEM) image of KP-SeNPs. **C** Selected area electron diffraction (SAED) image of KP-SeNPs. **D** X-ray analysis (XRD) spectrum of KP-SeNPs. **E** Energy-dispersive X-ray (EDX) analysis of KP-SeNPs. **F** Dynamic light scattering (DLS) spectrum of KP-SeNPs. **G** Fourier-transform infrared (FT-IR) spectrum of KP-SeNPs and KP-RE

Selenium nanoparticles (KP-SeNPs) were prepared using KP-RE as a reducing reagent for Na_2_SeO_3_. It was critical to determine the optimal concentrations of KP-RE and Na_2_SeO_3_ to ensure sufficiently low polydispersity of the SeNPs. Thus, different reaction parameters (concentrations of KP-RE and Na_2_SeO_3_, temperature, duration, and pH) were screened by optical density measurements using a UV–Vis spectrophotometer in the 350–750 nm wavelength range. According to the highest peak of the UV–Vis spectrum, the best quality KP-SeNPs were synthesized by the reaction of 2 mg/mL of KP-RE in a 2 mM Na_2_SeO_3_ solution at pH 9.0 and 80 °C for 3 h. To determine whether the SeNPs were synthesised successfully, UV–Vis absorption spectra of KP-RE and synthesized KP-SeNPs were compared (Fig. 1A). The synthesis of KP-SeNPs was confirmed by observing the change in color from purple to brown in the synthesis solution. As shown in Fig. 1A, the KP-RE gave rise to a wavelength of 550 nm. In contrast, KP-SeNPs showed a wavelength of 420 nm, suggesting the successful synthesis of KP-SeNPs. Consistent with our results, Sowndarya et al. reported a similar λmax for SeNPs [28]. Furthermore, we characterized the properties of KP-SeNPs using TEM, SAED, XRD, EDX, DLS, and FT-IR to determine whether KP-SeNPs were of high quality. First, the TEM images showed that the KP-SeNPs were spherical (Fig. 1B), and their SAED pattern (Fig. 1C), agrees well with the spherical character. XRD analysis indicated the space group and the crystallographic system. Our XRD results revealed that the SeNPs are crystalline. The pattern of XRD (Fig. 1D) shows two blunt peaks indexed to (100) and (202), indicating the Bragg’s reflection planes of a selenium lattice [29]. However, KP-SeNPs did not exhibit any sharp peaks, as the nanospherical sample was amorphous in character. Such SeNPs are also synthesized by many other plants as well as bacteria [30, 31]. In the EDX spectrum, the optical absorption peaks at 1.8 keV corresponded to selenium peak (Fig. 1E). Additional signals originating from copper and iron were also found in the EDX spectrum due to the grid used in EDX analysis. The quantitative analysis reveals the elemental composition of the samples, which is shown in Fig. 1E. Moreover, DLS analysis considers the organic shell when estimating the total size of conjugates in colloids or their average hydrodynamic sizes. The DLS analysis size range revealed that KP-SeNPs had a non-uniform distribution, with an average particle size of 213.6 nm (Fig. 1F).

FT-IR measurements were carried out to identify the signals present in KP-RE and KP-SeNPs, which are responsible for the reduction of Na_2_SeO_3_ by KP-RE. As shown in Fig. 1G, the peak for KP-RE at 3207.07 cm^−1^ corresponded to the hydroxyl functional group of phenolic compounds or alcohols [32]. C=C stretching in aromatic compounds or N–H groups of secondary amines was also detected at 1593.88 cm^−1^ which corresponds to a heterocyclic compound [33]. The peak at 1031.69 cm^−1^ is due to C–O stretching in alcohols [34]. Moreover, the C–N stretching vibrations of aliphatic and aromatic amines were determined at 1315.60 cm^−1^ from KP-SeNPs, with bands at 993.19 cm^−1^ being related to the C-H groups of aromatic compounds [35]. In contrast, the peak at 3264.65 cm^−1^ suggests a strong hydrogen bonding interaction between selenium and the O–H groups from KP-RE, facilitating the biosynthesis of SeNPs through the formation of Se O–H bonds [36]. The band at 2925.59 cm^−1^ is characteristic of the stretching vibration of the saturated aliphatic group, and is consistent with the KP-RE peak at 2925.29 cm^−1^ [37]. The peak detected at 1622.16 cm^−1^ represents the C=O stretch of carbonyl groups [38]. The weak absorption bands from KP-RE at 1392.79 cm^−1^ are related to C–H asymmetric bending in the CH_2_ and CH_3_ groups [39]. Together, these findings and peak changes suggest the impact of the functional groups of KP-RE as reducing and stabilizing agents in the synthesis of KP-SeNPs. We used *K. parviflora* and selenium salt to manufacture a new material, KP-SeNPs, with an average particle size of 214 nm, and the optimal manufacturing conditions were 2 mg/mL of KP-RE in 2 mM Na_2_SeO_3_ at pH 9.0, at 80 °C, for 3 h.

### Cytotoxic effects of KP- SeNP against normal and cancer cells

**Fig. 2:**
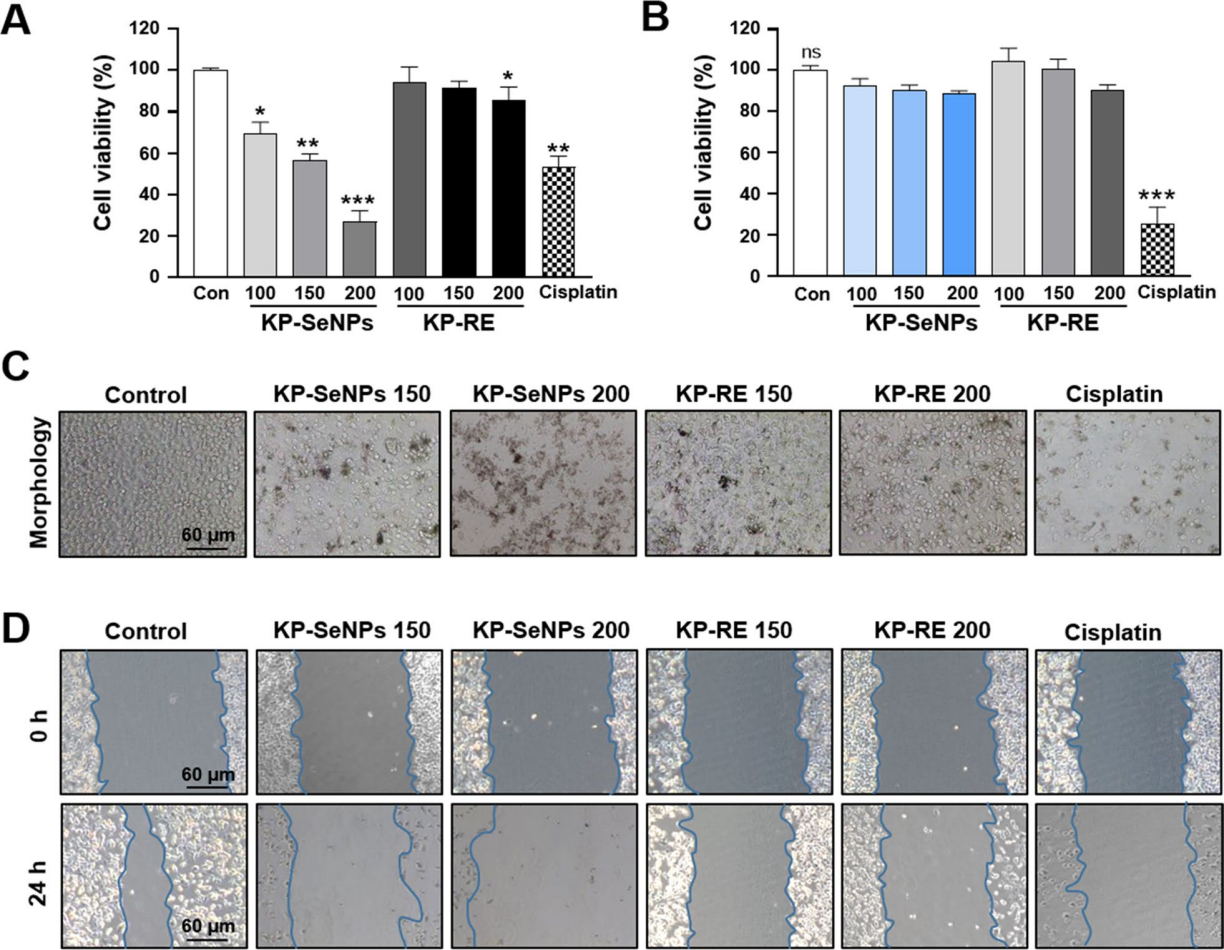
Cytotoxic effects of KP-SeNPs against cancer cells and normal cells. **A** The cytotoxic effects of KP-SeNPs (100, 150, 200 µg/mL) and KP-RE (100, 150, 200 µg/mL) on the AGS cells. Cisplatin (50 µM) was used as the positive control. **B** The cytotoxic effects of KP-SeNPs (100, 150, 200 µg/mL), KP-RE (100, 150, 200 µg/mL) and cisplatin (50 µM) against control HaCaT cells. **C**, **D** Microscopic images and wound-healing assay of AGS cells treated with KP-SeNPs (150, 200 µg/mL), KP-RE (150, 200 µg/mL), and Cisplatin (50 µM). *p < 0.05, ** *p* < 0.01, ***p < 0.001 compared with control

The cytotoxic effect of KP-SeNPs in AGS cells and HaCaT cells was evaluated through MTT assay. As shown in Fig. 2A and C, KP-RE did not exhibit strong cytotoxicity in AGS cells, whereas KP-SeNPs significantly decreased cell viability in a dose-dependent manner. Notably, 200 µg/mL of KP-SeNPs resulted in more significant inhibition of cell than the positive control (50 µM cisplatin). The wound-healing assays reflected the functional impact of KP-SeNPs on GC cell migration. Our results illustrated that KP-SeNPs treatment effectively inhibited the migration of AGS cells when compared with control groups (Fig. 2D). In the normal cells, although KP-SeNPs showed a slightly decreased viability, no considerable toxic activity was exerted at concentrations less than 200 μg/mL compared to that in control cells (Fig. 2B). In contrast, the positive control, cisplatin (50 μM) treatment, showed a significant toxic effect against HaCaT cells. These results indicated that KP-SeNPs cytotoxic for AGS cells without exerting significant toxic effects on normal cells. Based on these results, we suggest that KP-SeNPs could be a potential candidate in GC treatment with few side effects. Hence, we conducted a further investigation of the anticancer activities and signaling pathway mechanisms of KP-SeNPs.

### Mitochondrial damage-associated apoptosis induced by KP-SeNPs in AGS cells

**Fig. 3:**
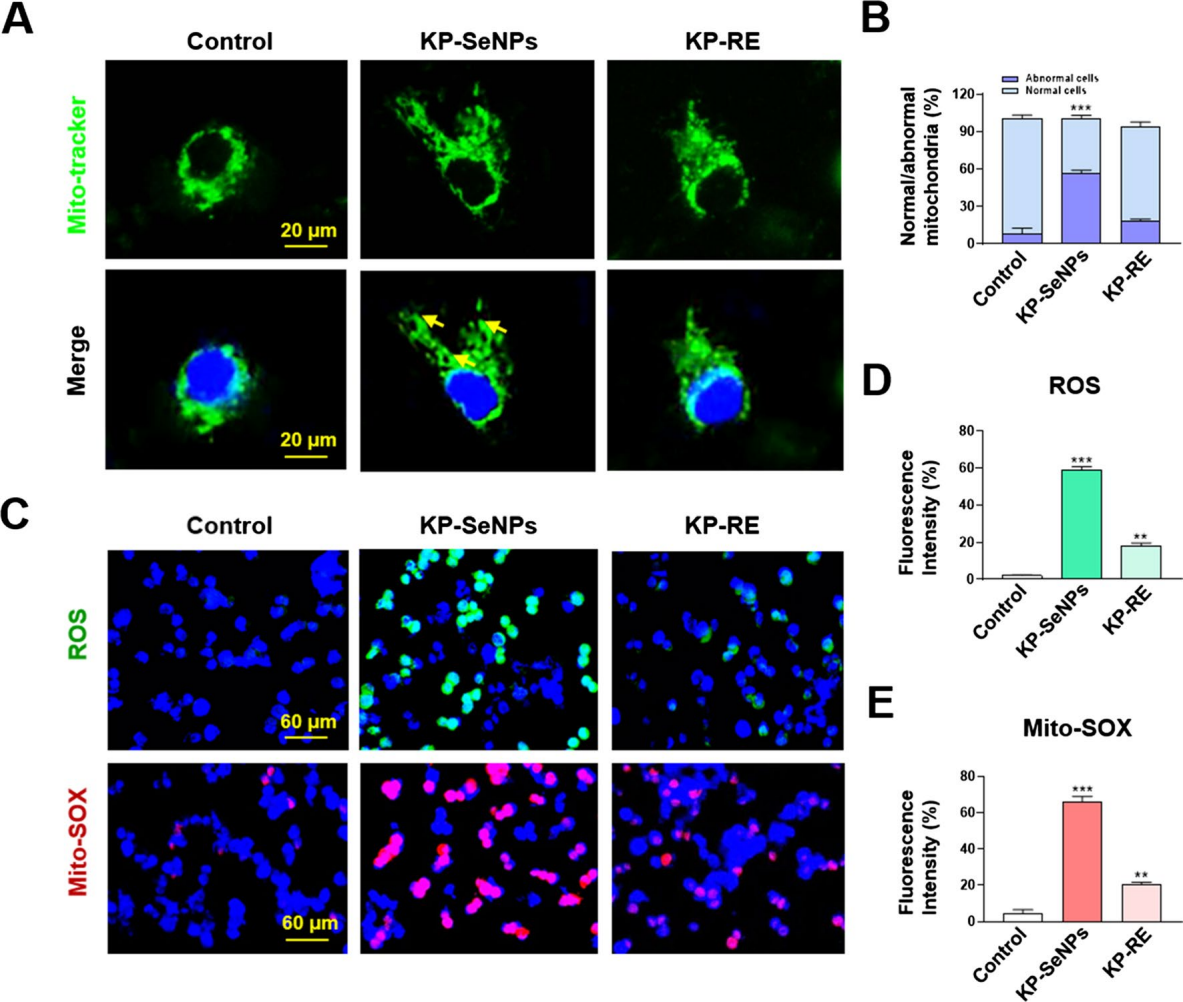
Mitochondrial damage induced by KP-SeNPs in AGS cells. **A** Mitochondrial morphology of AGS cells. The mitochondria showed a normal thread-like shape in the control group and the KP-RE-treated (200 µg/mL) group. The mitochondria showed abnormal lamellar shape or swollen round shape in KP-SeNP-treated (200 µg/mL) cells (yellow arrows). **B** Percentage of AGS cells with normal or abnormal mitochondrial morphology; n > 20. **C**–**E** Fluorescence microscopic images of mitochondrial reactive oxygen species (ROS) and mitochondrial superoxide (Mito-SOX) of KP-SeNPs (200 µg/mL) and KP-RE-treated (200 µg/mL) on AGS cells. The images were quantified by Image J software. ** *p* < 0.01, ***p < 0.001 compared with control

Given that KP-SeNPs have an anticancer effect on AGS cells, we explored the underlying mechanisms. Apoptosis, an important process in homeostasis, destroys abnormal cells including cancer cells. Indeed, many cancer therapies target the suppression of tumor cell proliferation by inducing apoptosis [40]. Mitochondrial impairment facilitates caspase activation, promoting apoptosis [41]. It has been proposed that mitochondrial dysfunction is caused by the loss of mitochondrial membrane potential (∆ψm) and the overproduction of reactive oxygen species (ROS) during caspase-induced apoptosis. The cleavage product of activated caspase 3 can directly induce apoptosis and regulate the B-cell lymphoma (Bcl) family proteins, including anti-apoptotic Bcl-2 and pro-apoptotic Bcl-associated X protein (Bax) [42, 43]. Additionally, it can promote caspase activation and apoptosis further through its cleavage product [41]. Fluorescence microscopy was used to examine the mitochondrial morphology of AGS cells treated with KP-SeNPs and KP-RE after staining with MitoTracker. As shown in Fig. 3A and B, swollen or lamella-shaped mitochondria (yellow arrow) were observed only in KP-SeNP-treated cells, whereas control cells and KP-RE-treated cells displayed typical tubular mitochondria, suggesting that mitochondrial damage occurs during KP-SeNP treatment [44] and induces mitochondrial impairment by regulating mitochondrial fusion. Moreover, excessive ROS and Mito-SOX (mitochondrial ROS) production generally lead to mitochondrial dysfunction [45]. Therefore, we measured ROS and Mito-SOX production. Compared to the control group, the production of ROS and Mito-SOX in AGS cells treated with KP-SeNPs were significantly increased, by 8.4 and 5.8 times, respectively (Fig. 3C–E). These results indicate that the apoptosis of KP-SeNP-treated AGS cells might be related to the excessive accumulation of ROS-induced mitochondrial damage. Interestingly, slight mitochondrial dysfunction such as the increase of ROS/Mito-SOX production was detected in the KP-RE-treated group, but it did not lead to cell death in AGS cells. It could be that ROS was induced by KP-RE but did not lead to imbalance in the AGS cells.

**Fig. 4:**
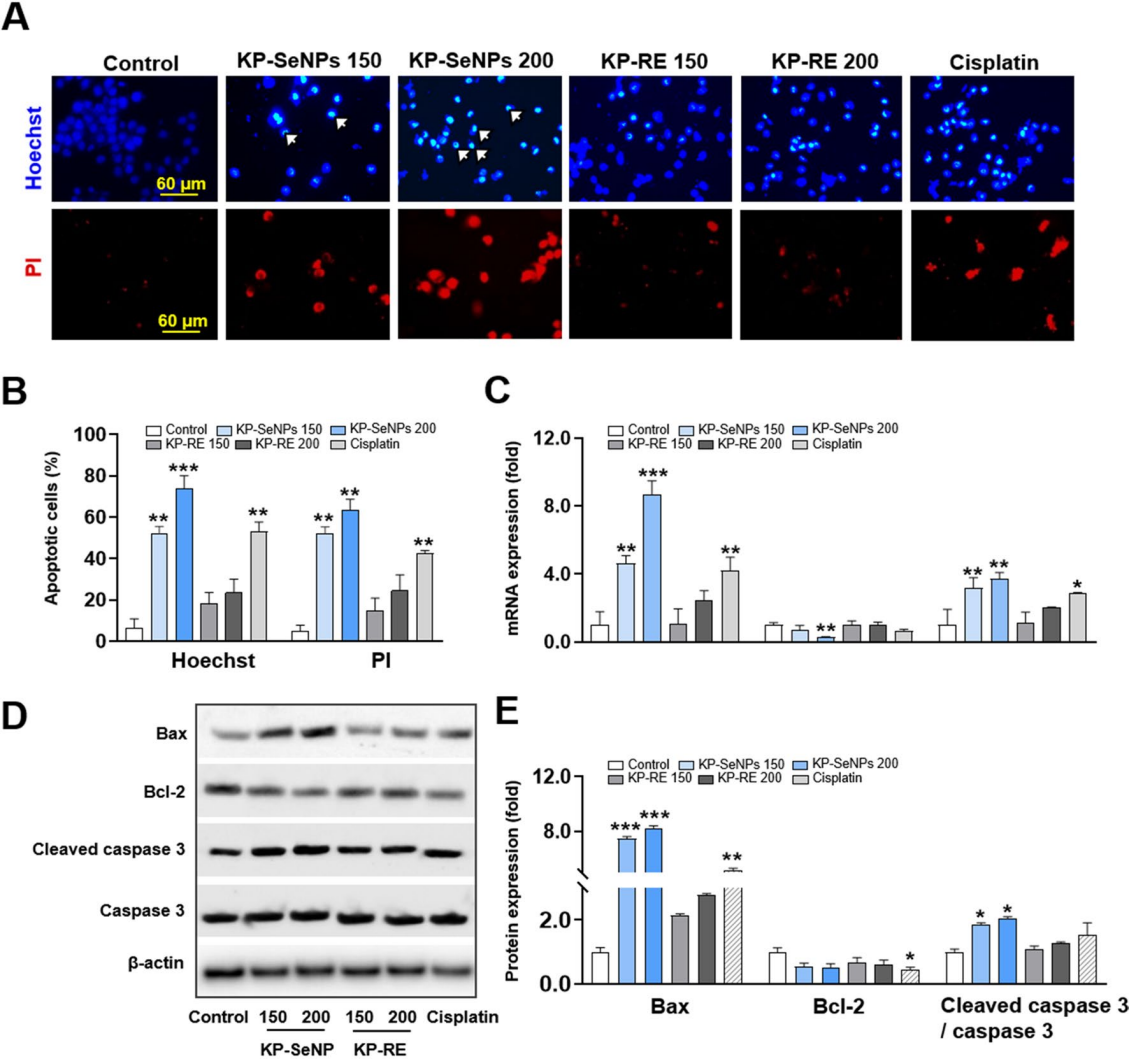
Apoptosis induced by KP-SeNPs in AGS cells. **A** Hoechst staining and propidium iodide (PI) staining images of AGS cells treated with KP-SeNPs (150, 200 µg/mL) and KP-RE (150, 200 µg/mL). Cisplatin (50 µM) was used as the positive control. The white arrows indicate fragmented nuclei with condensed chromatin. **B** Images were analyzed through the Image J software. **C** Effect of KP-SeNPs and KP-RE on the regulation of apoptosis-related gene expression in AGS cells. Cisplatin (50 µM) was used as the positive control. **D** Apoptosis-related protein expression visualized by western blotting. **E** Western blots were analyzed through Image J software. *p < 0.05, ** *p* < 0.01, ***p < 0.001 compared with control

Normally, the intrinsic apoptosis pathway of mammalian cells activates the Bcl-2 family and releases Bax to facilitate depletion of the mitochondrial outer membrane potential. Furthermore, apoptosis occurs through the release of caspase 3 and induction of cellular stress, such as elevated glucose concentrations or growth factor deprivation [46, 47]. Figure 4A and B show images of Hoechst and PI staining of AGS cells treated with KP-SeNPs, KP-RE, and cisplatin. As determined by Hoechst staining, the presence of faint blue nuclei in the control group showed that apoptosis had not occurred. KP-SeNPs (150 and 200 µg/mL) caused considerable fluorescence changes in the nuclei of the AGS cells, in contrast to the KP-RE-treated groups. Furthermore, PI staining showed that the extent of apoptosis in the KP-SeNP-treated cells was much greater than that in the cells of the KP-RE-treated group. These results suggest that, compared to KP-RE, KP-SeNPs considerably enhanced apoptosis in a dose-dependent manner. Next, we investigated the molecular mechanism responsible for the apoptosis triggered by KP-SeNPs. Compared to other apoptotic signaling pathways, the Bax/Bcl-2 signaling pathway is the most important in activating the mitochondrial apoptosis pathway, which increases the permeability of the mitochondrial membrane and triggers caspase 3 activation and subsequent cell death. First, we determined mRNA gene expression using qRT-PCR. As shown in Fig. 4C, KP-SeNPs significantly increased the gene expression of *Bax* and *Caspase 3* but decreased the expression of *Bcl-2* in AGS cells in a dose-dependent manner. Furthermore, the results of the intracellular protein expression analysis by western blotting (Fig. 4D, E) were consistent with the qRT-PCR results. This demonstrated that KP-SeNPs markedly enhanced the levels of apoptosis-related proteins, such as Bax/Bcl-2 and cleaved caspase 3/caspase 3. These findings indicate that mitochondrial impairment, induced by the activation of the intracellular apoptosis pathways via Bax/Bcl-2 and caspase 3, is likely to be the cause of death in KP-SeNP-treated AGS cells.

### KP-SeNP-induced autophagy in AGS cells

**Fig. 5:**
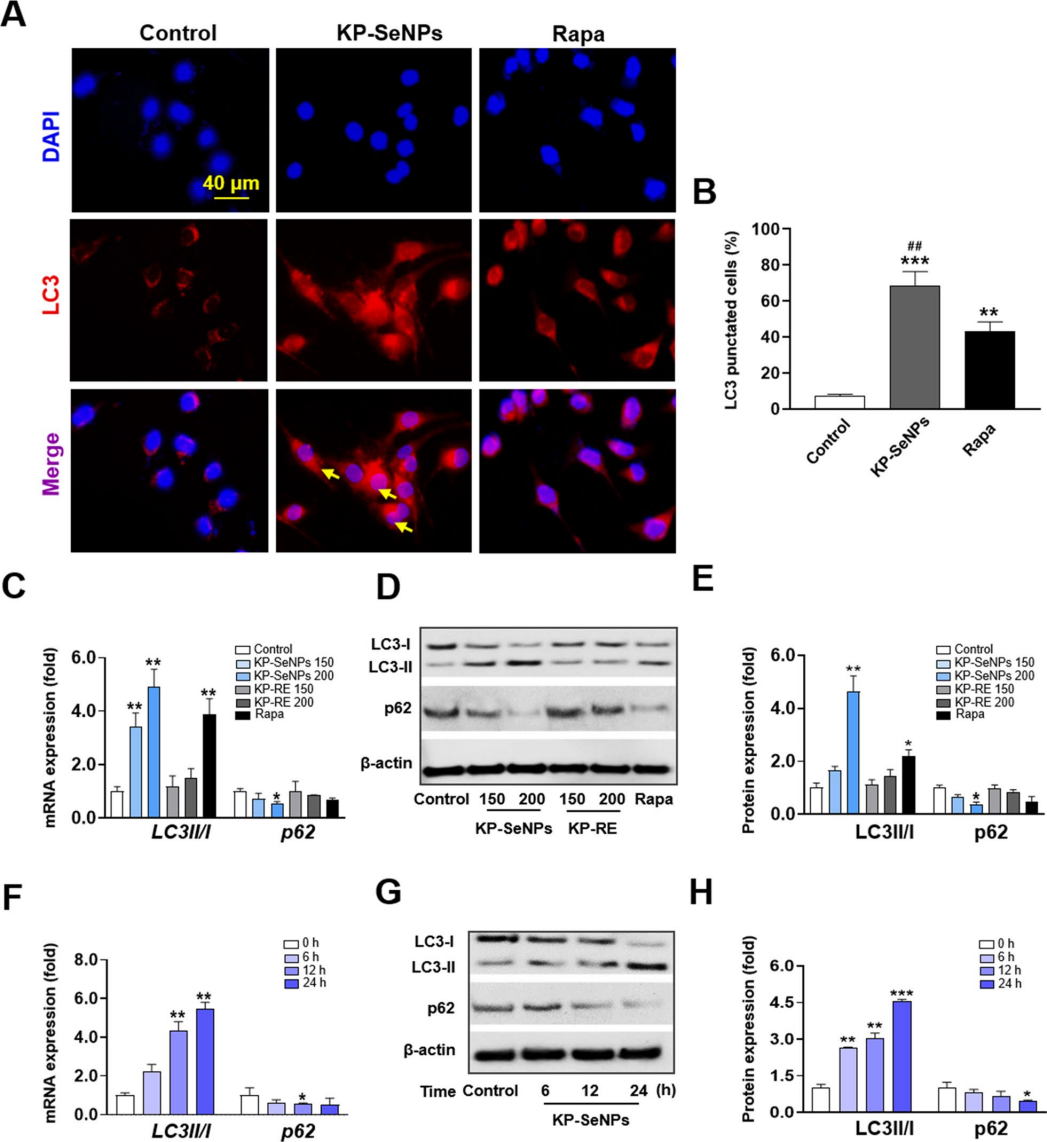
KP-SeNPs stimulates autophagy in vitro. **A** LC3 immunofluorescence staining of AGS cells treated with KP-SeNPs (200 µg/mL) and rapamycin (5 µM). **B** Quantification of the LC3 puncta average area from the images using ImageJ software. Quantification was performed from 3 experiments with n > 20 cells for each condition. Autophagy-related **C** mRNA and **D**, **E** proteins in AGS cells treated with KP-SeNPs (150, 200 µg/mL) and KP-RE (150, 200 µg/mL) for 24 h; Rapa (rapamycin, 5 µM) was used as the positive control. **F**–**H** AGS cells were treated with KP-SeNPs (200 µg/mL); mRNA and proteins were collected at the indicated time points and analyzed by qRT-PCR and western blot, respectively. The western blotting images were quantified through Image J software. *p < 0.05, ** *p* < 0.01, ***p < 0.001 compared with control

Apoptosis and autophagy are common tumor suppressor mechanisms. Apoptosis is a programmed cell-death pathway that prevents cancer cells from surviving. However, autophagy appears to have a dual role in cancer, since it has recently been demonstrated that autophagy aids tumor-cell survival in stressful situations, such as hypoxia or low-nutrient settings. Several studies have reported that excessive autophagy leads to cell death. Autophagy facilitates the breakdown of oncogenic chemicals, thereby inhibiting the growth of malignancies [48, 49]. Autophagy is characterized by several dynamic processes including induction, nucleation, elongation, maturation, and destruction. Among them, elongation is vital for complete autophagosome formation, and the shift from LC3-I to the next generation, LC3-II, is an important element in the process of elongation [50]. Thus, we first performed immunofluorescence labeling with an antibody against LC3 after treating AGS cells with KP-SeNPs (200 µg/mL) and rapamycin (5 µM, autophagy inducer). The images in Fig. 5A and B show that AGS cells in the control group exhibited a baseline level of autophagy, which is likely protective. Both the KP-SeNP- and rapamycin- treated groups showed an increase in the expression of LC3 when compared with the control group. The expression level of LC3 in cells treated with KP-SeNPs was higher than that in rapamycin-treated cells. This allowed us to preliminarily confirm that autophagy was activated in AGS cells. Moreover, the autophagy-related protein p62/SQSTM1 (p62) plays a critical role in autophagy as a cargo receptor for the deprivation of ubiquitinated substrates. When autophagy is initiated, the protein complex LC3-I is broken down into LC3-II, which is recruited to autophagosomes to interact with p62 (which mediates cargo selection), and total protein degradation proceeds via the formation of a synaptosome-lysosome complex [51]. Accordingly, degradation of p62 indicates the activation of autophagic flux. Therefore, the level of autophagy is frequently assessed by measuring the LC3-II/LC3-I ratio and p62 protein expression. Through qRT-PCR (Fig. 5C) and western blot analysis (Fig. 5D and E), we demonstrated that KP-SeNP-treatment-induced considerably higher levels of LC3-II in AGS cells, in a dose-dependent manner, compared to the control group and KP-RE groups. Simultaneously, a significant decrease in p62 protein expression was observed in the KP-SeNPs-treated group compared with that in the control and KP-RE groups. Moreover, 200 µg/mL of KP-SeNPs resulted in a greater increase in LC3II and inhibition of p62 than the positive control (5 µM rapamycin), suggesting the potential application of KP-SeNPs in autophagy-related cancer treatment. These data indicated that KP-SeNPs induced autophagy in human GC cells, to understand the dynamic shift of autophagy in AGS cells following KP-SeNPs treatment, we evaluated the expression levels of autophagic markers at different time points. From the results shown in Fig. 5F–H, we discovered that 6 h after KP-SeNPs treatment, an early autophagy event was initiated and that the activation of autophagic biomarkers (LC3-II/LC3-I and p62) lasted until 24 h, suggesting that 6–24 h post-treatment is the essential interval to activate autophagy in AGS cells. Thus, our findings indicate that KP-SeNPs may act as an anticancer drug in AGS cells by promoting autophagy.

### KP-SeNPs induce autophagy and apoptosis through the phosphatidylinositol 3-kinase (PI3K)/Akt/mTOR signaling pathway

**Fig. 6:**
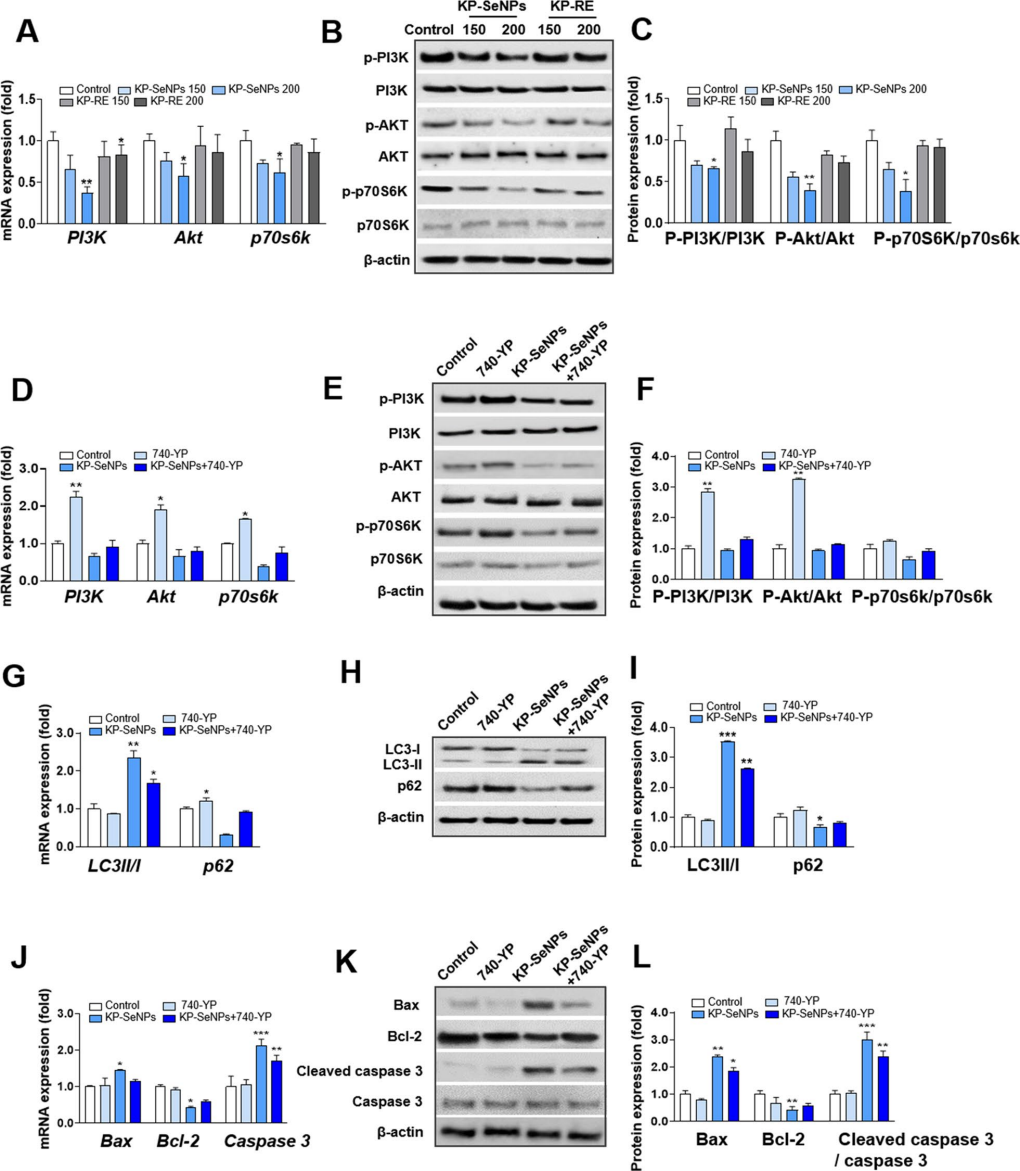
KP-SeNPs induce autophagy and apoptosis by inhibiting the PI3K/Akt/mTOR pathway. **A** Gene and protein **B**, **C** expression of PI3K/Akt/mTOR pathway markers and downstream effectors in AGS cells. Cells were treated with KP-SeNPs (150, 200 μg/mL) and KP-RE (150, 200 μg/mL) for 24 h. **D** Gene and protein (**E**, **F**) expression levels of PI3K/Akt/mTOR pathway markers and downstream effectors in AGS cells treated with KP-SeNPs (200 μg/mL), 740Y-P (30 μM), and KP-SeNPs (200 μg/mL) in combination with 30 μM 740Y-P for 24 h. KP-SeNPs promote autophagy through the PI3K/Akt pathway. The autophagy-related gene (**G**) and protein (**H**, **I**) expression levels of LC3 II/I and p62. KP-SeNPs induce apoptosis in AGS cells through the PI3K/Akt pathway. The apoptosis-related gene (**J**) and protein (**K**, **L**) expression of Bax, Bcl-2, and caspase 3. The images of western blots were measured using Image J. The asterisks in the graph represent significant differences between sample and control. *p < 0.05, **p < 0.01, ***p < 0.001, compared with control

Several studies have shown that the PI3K/Akt pathway is a representative regulator of growth, proliferation, cell cycle, metastasis, apoptosis, and autophagy [52–54]. The mechanistic target of rapamycin (mTOR) is a downstream target of the PI3K/Akt pathway that regulates p70S6K. The PI3K/AKT/mTOR signaling pathway is associated with autophagy and apoptosis and plays a vital role in both processes [55]. Inhibition of the mTOR pathway enhances the production of autophagosomes, which regulate cell survival and death [56]. Several anticancer drugs induce apoptosis and autophagy by inhibiting the PI3K/Akt/mTOR pathway [57–59]. There is also evidence of an interaction between autophagy and apoptosis through the Bcl-2 family and PI3K/Akt/mTOR signaling pathway [60]. Thus, to determine the detailed molecular mechanisms of KP-SeNPs and its anticancer effects in GC cells, we examined whether KP-SeNPs regulate autophagy and apoptosis through the PI3K/Akt/mTOR pathway. Figure 6A–C show that compared with the control group, gene expression and phosphorylation of PI3K or Akt decreased in a dose-dependent manner after 24 h of treatment with KP-SeNPs, but KP-RE did not show obvious pathway inhibition. In addition, we found that KP-SeNPs treatment of AGS cells suppressed the gene expression levels of mTOR downstream effector p70S6K, and decreased the activated form of it. Based on these results, we hypothesized that KP-SeNPs inhibit the PI3K/Akt/mTOR pathway. Furthermore, to explore whether the PI3K/Akt/mTOR pathway is the key to inducing autophagy and apoptosis in KP-SeNP-treated AGS cells, we determined the expression of autophagy- and apoptosis-related genes and proteins using the PI3K activator, 740Y-P. According to our results (Fig. 6D–F), co-treatment with KP-SeNPs and 740Y-P inhibited the phosphorylation of PI3K, Akt, and 70S6K, compared to 740Y-P-treatment alone, suggesting that KP-SeNPs promote the suppression of the PI3K/Akt/mTOR signaling pathway. Moreover, KP-SeNP-induced autophagy was attenuated by pre-treatment with 740Y-P, as evidenced by increased expression of the autophagy marker p62 and downregulation of the autophagy marker LC3-II (Fig. 6G–I). Meanwhile, co-treatment with 740Y-P and KP-SeNPs upregulated the apoptosis factor Bcl-2, but downregulated the apoptosis factors Bax and caspase 3, compared with the KP-SeNP-treatment group (Fig. 6J–L). Taken together, these findings show that KP-SeNPs induce autophagy and apoptosis protein expression through the downregulation of the PI3K/Akt/mTOR pathway.

### Autophagy is a driving mechanism for the KP-SeNPs induction of apoptosis

**Fig. 7:**
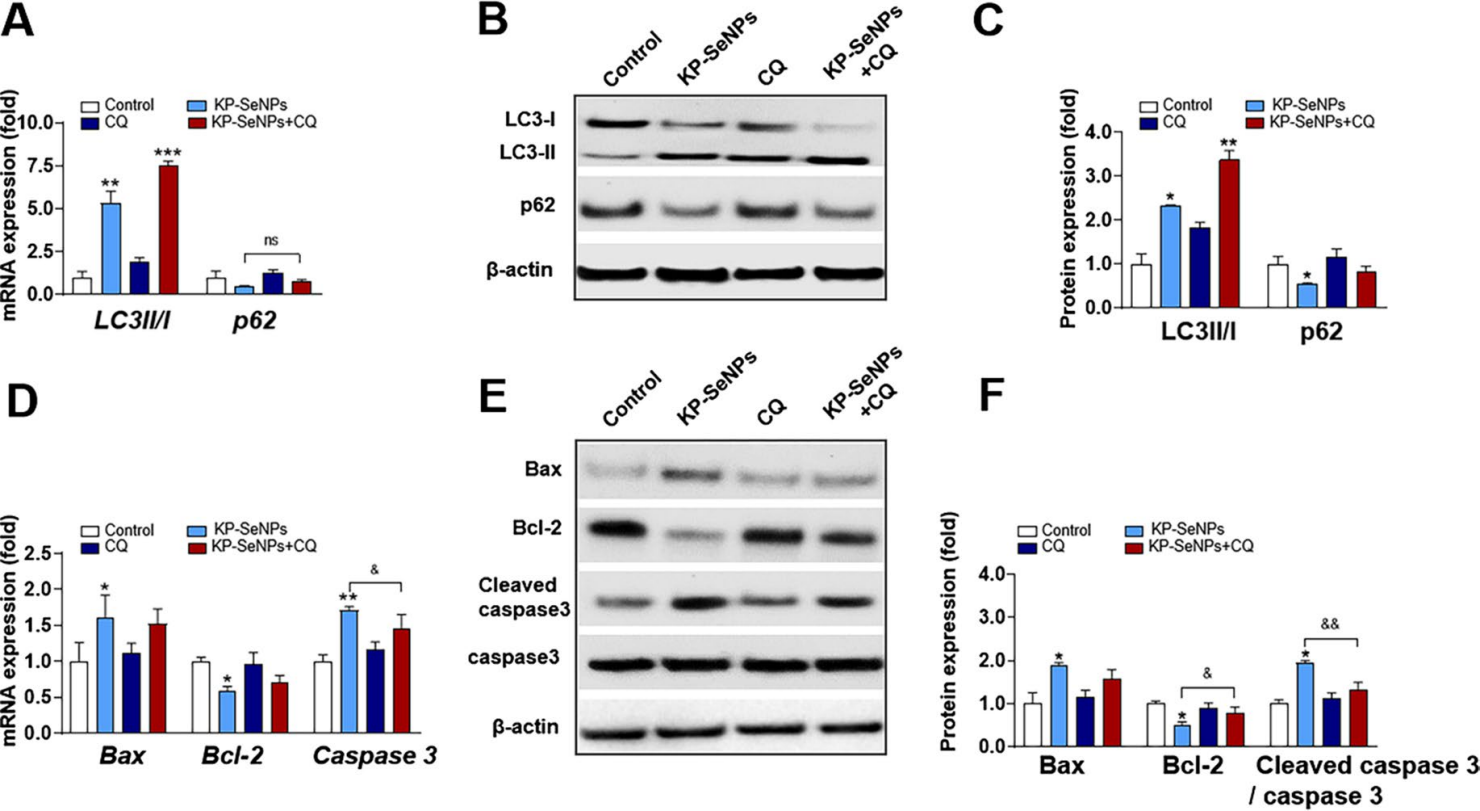
KP-SeNPs promote apoptosis by inducing autophagy. The autophagy inhibitor chloroquine (CQ, 30 μM) was used to detect the effect of KP-SeNPs (200 μg/mL) on the association between autophagy and cytotoxicity in AGS cells. AGS cells were treated with KP-SeNPs (200 μg/mL) for 24 h in the presence or absence of 30 μM CQ. Autophagy-related gene (**A**) and protein (**B**, **C**) expression levels of LC3 II/I and p62. The apoptosis-related gene (**D**) and protein (**E**, **F**) expression levels of Bax, Bcl-2, cleaved caspase 3, and caspase 3. *p < 0.05, ** *p* < 0.01, ***p < 0.001, compared with control; ^&^*p* < 0.05, ^&&^ p < 0.01, KP-SeNPs compared with KP-SeNPs + CQ

In recent years, autophagy has become a target for anticancer therapy [61]. We showed that AGS cells treated with KP-SeNPs have enhanced autophagy. Next, we studied the involvement of autophagy in KP-SeNP-induced apoptotic cell death in AGS cells using the autophagy inhibitor chloroquine (CQ). Usually, CQ prevents autophagosomes from combining with lysosomes, thereby causing the accumulation of degraded proteins in cells and blocking autophagy. Our results established that inhibition of autophagy has a protective effect through an anti-apoptotic response. Specifically, we found that treatment with KP-SeNPs and CQ both could increase LC3-II mRNA and protein expression compared with the control group, whereas co-treatment with CQ and KP-SeNPs significantly raised LC3-II expression (Fig. 7A–C). Conversely, only CQ treatment increased the gene expression and protein levels of p62 in AGS cells, whereas co-treatment with KP-SeNPs and CQ decreased the gene expression and protein levels of p62. These results suggest that the decrease in autophagic flux caused by CQ could be reversed by treatment with KP-SeNPs. Additionally, in comparison to the KP-SeNPs group, co-treatment with CQ and KP-SeNPs lowered the expression of *Bax* and *Caspase3* mRNA and their corresponding protein levels and increased the expression of Bcl-2 (Fig. 7D–F), suggesting that apoptosis was blocked by the inhibition of autophagy. Overall, through the inhibition of autophagy induced by KP-SeNPs, we concluded that KP-SeNPs may contribute to AGS cell suppression via the PI3K/Akt/mTOR signaling pathway, indicating that autophagy is a critical early step in KP-SeNP-mediated AGS cell suppression.

### KP-SeNPs have an anti-tumor effect in AGS-xenograft mice

**Fig. 8:**
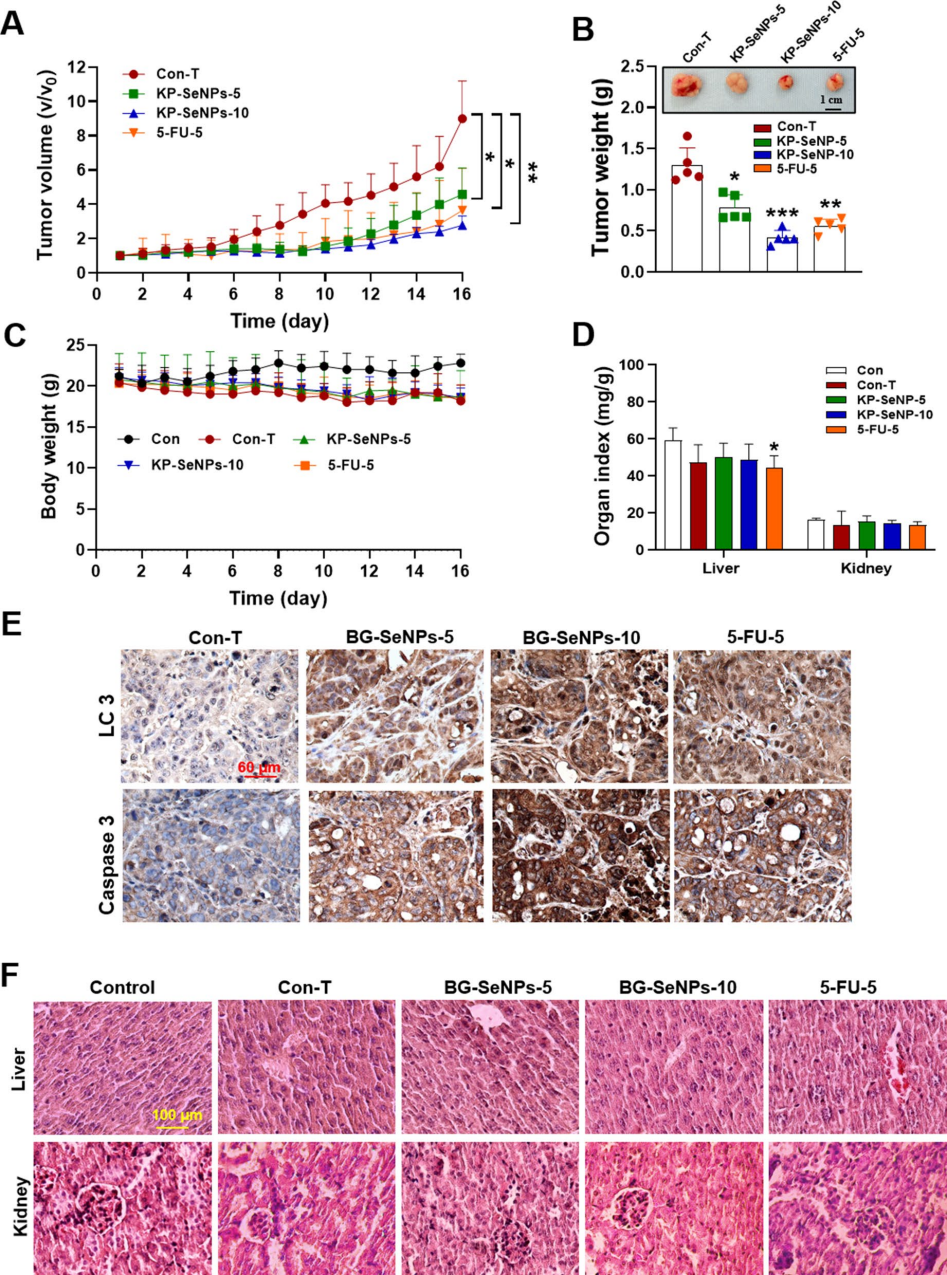
KP-SeNPs have in vivo anticancer effects. KP-SeNPs and 5-FU were administered to AGS xenograft mice (n = 5) for 16 days. **A** Volume of tumor tissues isolated from xenograft-bearing mice. **B** Tumor weight and size in AGS xenograft-bearing mice. **C** Body weights of the xenograft-bearing mice. **D** Weight indices of the liver and kidneys. **E** IHC staining of LC3 and caspase 3 in tumor tissues isolated from AGS xenograft-bearing mice. **F** Hematoxylin and eosin Y staining of the liver and kidneys of mice from each group. *p < 0.05, **p < 0.01, and ***p < 0.001 compared with Con-T. Abbreviations: Con: Control; Con-T: Con-tumor; KP-SeNP-5: 5 mg/kg of KP-SeNPs; KP-SeNP-10: 10 mg/kg of KP-SeNPs; 5-FU-5: 5 mg/kg of 5-FU

To investigate the anticancer effect in vivo, KP-SeNPs were orally administered to thymus-deficient nude mice with AGS xenografts. To test the anticancer and biosafety capabilities of KP-SeNPs, 5-FU was supplied to the mice as a positive control. According to the results shown in Fig. 8A, B, compared to the control tumor (Con-T) group mice, daily treatment with KP-SeNPs and 5-FU considerably decreased tumor progression when visually evaluated. In comparison to the Con-T group, tumor sizes and weights in mice fed a high concentration of KP-SeNPs (10 mg/kg were reduced by 69.18% and 67.82%, respectively. Moreover, the groups receiving KP-SeNPs at a dosage of 10 mg/kg displayed greater suppression of tumor size, weight, and volume than mice that were administered only 5-FU. As shown in Fig. 8C, during the complete treatment period, no significant difference was observed in body weight between the Con-T, the KP-SeNP-treated, or the 5-FU-treated groups. As shown in Fig. 8D, compared with the control group, the KP-SeNPs treatment group showed a slight decrease in liver and kidney weight after the 16-day administration. Remarkably, the mice in the 5-FU group showed a significant decrease in liver and kidney weights compared to those in the Con-T group.

The enhanced inhibitory effect of KP-SeNPs on tumor growth was further supported by IHC staining. As shown in Fig. 8E, AGS xenograft tumors stained for caspase 3 and LC3 by IHC showed diffuse brown staining in the cytoplasm with very fine pale, speckled structures. By contrast, in the KP-SeNP-treated group, covalent binding of HRP to LC3 and caspase3 was detected, followed by substantial signal amplification. In KP-SeNP-treated AGS xenograft sections, LC3 and caspase 3 were detected as dark punctate structures in the cytoplasm of live tumors, and the treated tumors had larger vacuoles, reflecting some degree of necrosis. Interestingly, upregulation of LC3II and caspase 3 proteins was also found in the tumor tissues of 5-FU-treated mice. This is consistent with a study by Yang et al., which showed that 5-FU may exert an inhibitory effect on GC cell proliferation by inducing autophagy and specific autophagic cell death [62]. These results suggest that KP-SeNPs treatment leads to an increase in the levels of activated LC3 and caspase 3 in tumor cells, leading to autophagy and apoptosis to inhibit tumor growth. In addition, compared with the Con-T group, no pathological phenomena, such as cell edema and organelle disintegration, occurred in the KP-SeNP-treated groups (Fig. 8F). The structures surrounding the blood vessels can be observed in the tissue. Except for the increased infiltration of inflammatory cells in the central area of the liver tissue in the 5-FU group, there was no obvious histological damage to the liver and kidney in the other groups, which confirmed the low toxicity of KP-SeNPs in vivo. Based on the above results, we concluded that KP-SeNPs have a good anti-cancer effect without causing obvious liver and kidney damage.

### Effects of KP-SeNPs treatment on the safety of mice

**Fig. 9:**
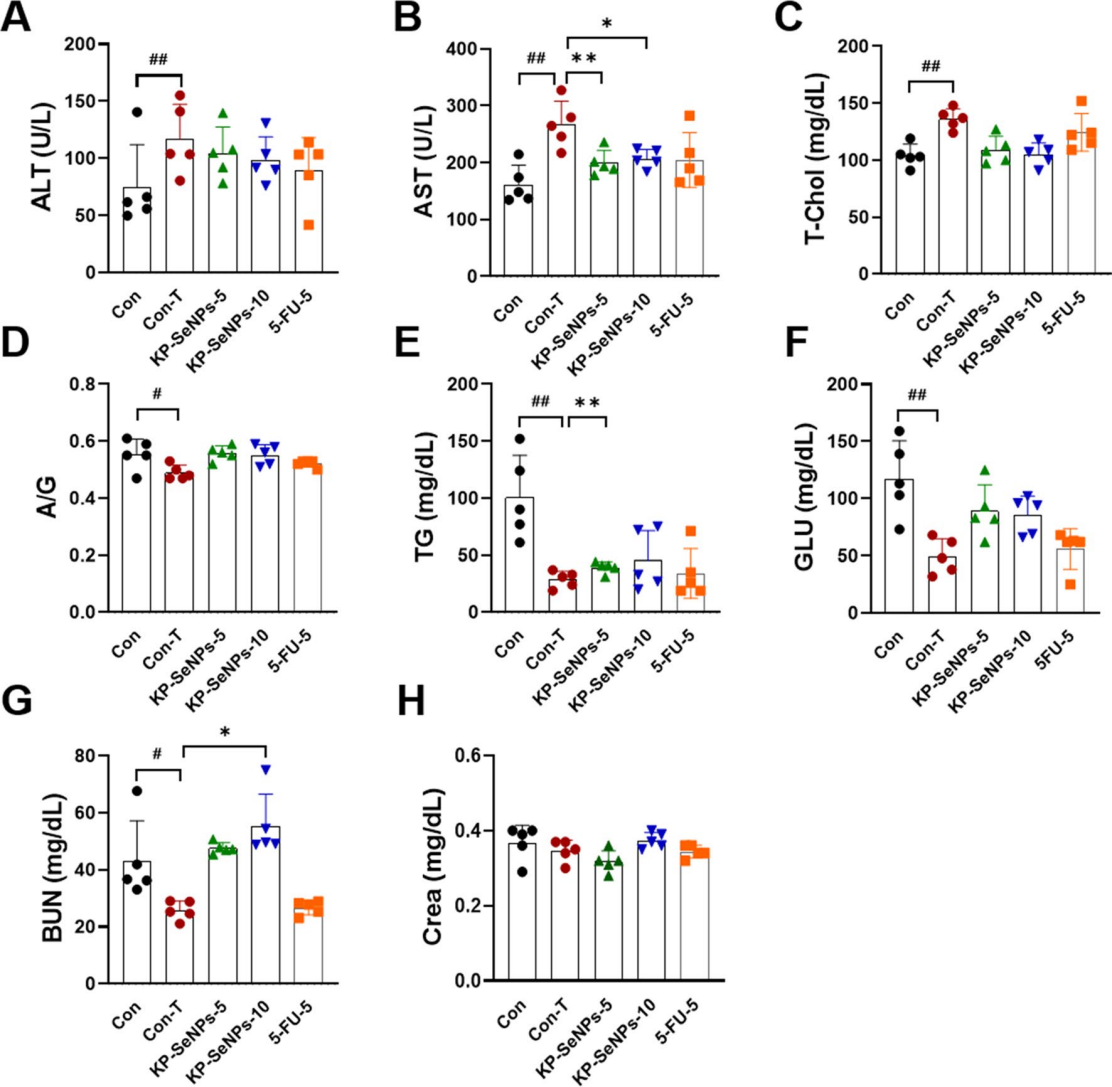
Serum biochemical analysis in KP-SeNPs administration in AGS xenograft-bearing mice. KP-SeNPs (5 and 10 mg/kg) treatment was assayed using 5-FU (5 mg/kg) treatment as a positive control. **A** Alanine aminotransferase (ALT); **B** Aspartate aminotransferase (AST); **C** Total cholesterol (T-Chol); **D** Albumin/globulin (A/G) ratio; **E** triglyceride (TG); **F** Glucose (GLU); **G** Blood urea nitrogen (BUN); and **H** Creatinine (Crea) were measured. *p < 0.05, **p < 0.01 compared with Con-T. ^#^*p* < 0.05, ^##^*p* < 0.01 Con-T compared with Con group

The hepatobiliary and renal systems are the main routes through which drugs and their metabolites leave the body. Drug-induced liver and kidney injury is the leading reason for the termination of drug discovery research projects [63]. Although the excellent selective toxicity of KP-SeNPs in cancers has been demonstrated in vitro and in vivo, we continued to test blood chemistry indices (such as ALT, AST, T-Chol, A/G, TG, GLU, BUN, and Crea levels) to further assess the toxic effects of KP-SeNPs. ALT and AST levels are indicators for proper liver function. Damage to the liver tissue may lead to a disturbance in the amount of these enzymes secreted into the bloodstream. As shown in Fig. 9A–F, the Con-T group had higher ALT, AST, and T-Chol levels than the Con group, but lower A/G ratio, TG, and GLU levels. KP-SeNPs substantially reduced the AST content, ALT and T-Chol levels compared to that in the Con-T group. Low-dose KP-SeNPs treatment enhanced GLU compared to high-dose KP-SeNPs administration. These results indicate that the AGS-xenograft tumor influenced liver function in mice, but KP-SeNPs may have a protective effect in the liver. There were no statistically significant differences in these biochemical parameters between the 5-FU and Con-T groups. Moreover, the results of all mice renal function tests, including blood urea nitrogen (BUN) and (creatinine) Crea tests, were compared. The results (Fig. 9G, H) indicated that the BUN levels of Con-T group are significantly lower than those of the control group, which was restored by KP-SeNPs treatment. The Crea levels of the KP-SeNP-treated mice did not differ significantly from those of the control group. Taken together, our findings demonstrate that, the KP-SeNPs treatment groups, tumor growth was satisfactorily inhibited with good biosafety. However, the biodistribution of KP-SeNPs after administration and the safety of KP-SeNPs for clinical uses need to be addressed before their clinical transformations.

### Analysis of the bioactive components in KP-SeNPs by LC–MS

The results of in vitro and in vivo experiments indicated KP-SeNPs had higher anticancer activities than black ginger ethanol extract (KP-RE). Therefore, we endeavored to detect the bioactive components that contributed to the anticancer effects of KP-SeNPs. The chromatogram of the total positive ion current showed five main peaks for KP-SeNPs (Additional file 1: Fig. S1). As shown in Table 1, tetramethoxyflavone, quercetin pentamethyl ether, dimethoxyflavone, trimethoxyflavone, and trihydroxy-dimethoxyflavone were identified in KP-SeNPs through the confirmation of retention times (13.372, 14.096, 14.228, 14.497, and 15.339 min, respectively). Among these compounds, tetramethoxyflavone, dimethoxyflavone, and trimethoxyflavone, which are abundant in natural sources, have a significant anticancer activity [64–66]. In addition to these two compounds, quercetin-pentamethyl ether and trihydroxy-dimethoxyflavone were also present in KP-SeNPs. Quercetin-pentamethyl ether was found at its highest peak in KP-SeNPs, which showed sirtuin-activating and anti-glycation activities in a previous report [67], and trihydroxy-dimethoxyflavone showed potent anti-inflammatory activity without any observable cytotoxicity [68]. Thus, we speculate that these two compositions may contribute to the good biosafety of KP-SeNPs. Taken together, these observations indicate that tetramethoxyflavone, dimethoxyflavone, and trimethoxyflavone may be considered anticarcinogenic and thereby may contribute to the anticancer activity of KP-SeNPs. However, further investigation is necessary to make sure which element is responsible for such anticancer activity, since the KP-RE contains other elements which are highly effective in biology. And the use of these components in combination with other nanoplatforms may help develop more effective therapeutic approaches for cancer.

**Table 1:** Main components in KP-SeNPs

No	RT (min)	Compound	Formula
1	13.372	Tetramethoxyflavone	C_19_H_18_O_6_
2	14.069	Quercetin-pentamethyl Ether	C_20_H_20_O_7_
3	14.228	Dimethoxyflavone	C_17_H_14_O_4_
4	14.497	Trimethoxyflavone	C_18_H_16_O_5_
5	15.339	Trihydroxy-dimethoxyflavone	C_17_H_14_O_7_

## Conclusion

In this study, *K. parviflora* (black ginger) root extract was successfully harvested using ecofriendly selenium nanoparticles for enhanced anti-GC therapy. The optimum conditions for synthesizing KP-SeNPs were determined by the reaction of 2 mg/mL of KP-RE in a 2 mM Na_2_SeO_3_ solution at pH 9.0 and 80 °C for 3 h. In vitro investigations confirmed that enhancing autophagy induces the apoptotic effects of KP-SeNPs, specifically through the inhibition of PI3K/Akt/mTOR, and that KP-SeNPs are a potential therapeutic target for achieving cellular anticancer effects. Moreover, in vivo studies further demonstrated that KP-SeNPs exhibited effective cytotoxicity against AGS cancer cells with good biosafety. Finally, we identified five potential bioactive components in KP-SeNPs which may contribute to the anticancer activity of KP-SeNPs. Overall, our study presents KP-SeNPs as an ideal candidate for the development of selenium-nanoparticle based in the field of anticancer therapy.

## Supplementary Information

**Additional file 1: Table S1.** Real-time PCR primers used in the qRT-PCR assays. **Figure S1.** Liquid chromatography-mass spectrometry (LC-MS) analysis of KP-SeNP.

## Data Availability

The authors confirm that the data supporting the findings of this study are available within the article.

## Abbreviations

GC: Gastric cancer
SeNPs: Selenium nanoparticles
PC-3: Human prostate cancer cells
SKOV3: Human ovarian cancer cells
A549: Human lung adenocarcinoma
KP-SeNPs: *Kaempferia parviflora* Selenium nanoparticles
KP-RE: Kaempferia parviflora root extract
PI3K: Phosphoinositide 3-kinase
mTOR: Mammalian target of rapamycin
FBS: Fetal bovine serum
PS: Penicillin/streptomycin
PVDF: Polyvinylidene fluoride
ECL: Enhanced chemiluminescence
Na2SeO3: Sodium selenite
MTT: 3-(4,5-Dimethylthiazol-2-yl)-2,5-diphenyltetrazolium bromide
PI: Propidium iodide
RT: Room temperature
TEM: Transmission electron microscopy
SAED: Selected area electron diffraction
EDX: Energy Dispersive X-ray
XRD: X-ray diffraction
DLS: Dynamic light scattering
FTIR: Fourier-transform infrared
AGS: Adenocarcinoma gastric cell line
HaCaT: Human keratinocyte cell line
KCLB: Korean Cell Line Bank
Cis: Cisplatin
LC3: Microtubule-associated protein 1A/1B-light chain 3
Rapa: Rapamycin
FITC: Fluorescein isothiocyanate
IHC: Immunohistochemical
Caspase 3: Cysteine-aspartic acid protease 3
Bcl-2: B-cell lymphoma 2
Bax: Bcl-2-associated X protein
p62: P62/SQSTM1
p70s6k: Ribosomal protein S6 kinase beta-1 (S6K1)
5-FU: 5-Fluorouracil
H&E: Hematoxylin and eosin Y
ALT: Alanine aminotransferase
AST: Aspartate aminotransferase
T-Chol: Total cholesterol
A/G: Albumin/globulin
TG: Triglyceride
GLU: Glucose
BUN: Blood urea nitrogen
Crea: Creatinine
LC–MS: Liquid chromatography–mass spectrometry
