# Supplementary material for: Biologically synthesized black ginger-selenium nanoparticle induces apoptosis and autophagy of AGS gastric cancer cells by suppressing the PI3K/Akt/mTOR signaling pathway
Source: J Nanobiotechnology. 2022 Oct 8;20:441. doi: 10.1186/s12951-022-01576-6 (PMC9548198; doi:10.1186/s12951-022-01576-6)

Table S1. Real-time PCR primers used in the qRT-PCR assays.

| **Target gene** | **Forward Primer sequence (5’-3’)** | **Reverse Primer sequence (5’-3’)** |
| --- | --- | --- |
| *Bax* | AGCAAACTGGTGCTCAAGGC | CCACAAAGATGGTCACTGTC |
| *Bcl-2* | GTGGTGGAGGAACTCTTCAG | GTTCCACAAAGGCATCCCAG |
| *Caspase 3* | CCTCAGAGAGAGACATTCATG | GCAGTAGTCGCCTCTGAAG |
| *LC3-I* | ACATGAGCGAGTTGGTCAAGATCA | GATGGATTCTGGCCCAGTCATATT |
| *LC3-II* | ATAATTAGAAGGCGCTTACAGCTC | TGGCAGGTTCTCTTCTCTAGATCT |
| *SQSTM1(p62)* | ACCTGTCTGAGGGCTTCTCGCACA | CTCTTCTCCTCTGTGCTGGAACTC |
| *PI3K* | AGCTGTGGATCTTAGGGACCTCA | ACCATGACTGTGTACCAGAACAA |
| *AKT* | TACTACGCCATGAAGATCCTCAA | TCTGGAAAGAGTACTTCAGGGCT |
| *p70S6K* | CTCCCTACCTCACACAAGAAGCTC | GAAGTTCTTCCCAGTTAATGTGTC |
| *ꞵ-actin* | TCACCAACTGGGACGACAT | CACAGCCT GGATAGCAACG |

Figure S1. Liquid chromatography-mass spectrometry (LC-MS) analysis of KP-SeNP.


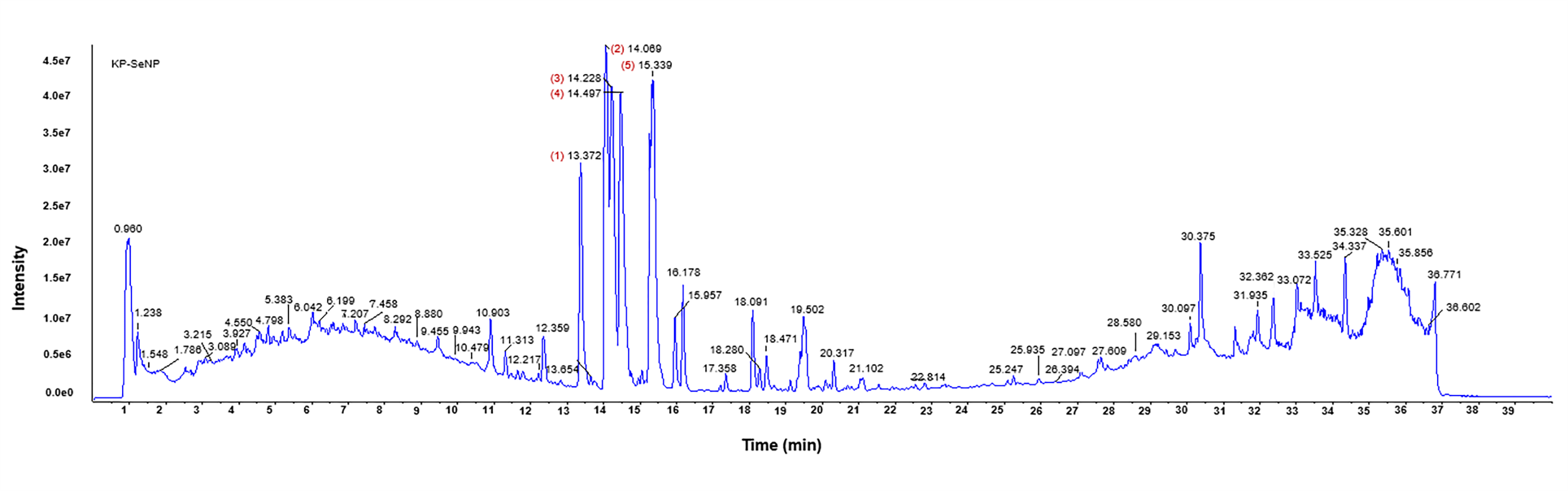

Supplement: Supplementary file 1 — Additional file 1: Table S1. Real-time PCR primers used in the qRT-PCR assays. Figure S1. Liquid chromatography-mass spectrometry (LC-MS) analysis of KP-SeNP. [file 12951_2022_1576_MOESM1_ESM.docx]
